# Advances in the understanding and clinical management of mastocytosis and clonal mast cell activation syndromes

**DOI:** 10.12688/f1000research.9565.1

**Published:** 2016-11-14

**Authors:** David González-de-Olano, Almudena Matito, Alberto Orfao, Luis Escribano

**Affiliations:** 1 Department of Allergy, Hospital Universitario Ramón y Cajal, Madrid, 28034, Spain; 2 Instituto de Estudios de Mastocitosis de Castilla La Mancha (CLMast), Hospital Virgen del Valle, Toledo, 45071, Spain; 3 Centro de Investigación del Cáncer/IBMCC (CSIC/USAL), Departamento de Medicina, IBSAL and Servicio General de Citometría, University of Salamanca, Salamanca, 37007, Spain

**Keywords:** Management, Mast Cell Activation Syndromes, Mastocytosis, Treatment

## Abstract

Clonal mast cell activation syndromes and indolent systemic mastocytosis without skin involvement are two emerging entities that sometimes might be clinically difficult to distinguish, and they involve a great challenge for the physician from both a diagnostic and a therapeutic point of view. Furthermore, final diagnosis of both entities requires a bone marrow study; it is recommended that this be done in reference centers. In this article, we address the current consensus and guidelines for the suspicion, diagnosis, classification, treatment, and management of these two entities.

## Introduction

Mast cells (MCs) are a key structural and functional component of the immune system and play a key role in inflammatory reactions, and at the same time they are the main effector cells in allergic processes ^1– 3^. MC disorders might present with a great variety of clinical symptoms or signs, such as skin involvement, which might lead to suspicion of the disease. Nevertheless, among other MC disorders, two entities frequently represent a diagnostic and therapeutic challenge in routine clinical practice in allergy: (i) indolent systemic mastocytosis presenting without skin involvement (ISMs ^−^) and (ii) clonal mast cell activation syndrome (c-MCAS). Of note, both entities are closely related to anaphylaxis, and their diagnosis requires specific techniques. Here, we review the current consensus and guidelines for the diagnosis, classification, treatment, and management of these two entities.

## Mastocytosis and other mast cell disorders: definition and classification

### Mastocytosis

The term systemic mastocytosis (SM) is used to define a heterogeneous group of rare diseases characterized by the presence of abnormal MCs in various organs and tissues ^4^. Two critical biological findings which are linked to the pathogenesis of the disease have been described: (i) activating somatic mutations in the *KIT* gene (usually the *KIT* Asp816Val D816V mutation) and the presence of an aberrant immunophenotype associated with the expression of CD25 on (bone marrow [BM]) clonal MCs. The current World Health Organization (WHO) classification of the disease includes up to seven distinct categories that meet the diagnostic criteria for mastocytosis ( Table 1). However, the development of new, more sensitive and specific methods, such as multi-parameter flow cytometry and highly sensitive polymerase chain reaction (PCR)-based techniques for the detection of aberrant MCs present at very low frequencies ^5– 8^ and the study of the *KIT* mutation in purified cells ^9^ or blood ^10– 12^ or both, have led to an unprecedentedly increased rate of detection of phenotypically aberrant and *KIT* mutated MCs in BM and peripheral blood, pointing out not only the potential need to revise current diagnostic and classification criteria to recognize new entities with very low tumor burden associated with life-threatening symptoms such as anaphylaxis but also a potential impact on the long-term prognosis of patients with indolent forms of the disease.

**Table 1.** World Health Organization 2016 criteria for the diagnosis and classification of systemic mastocytosis ^4^.

Classification of mastocytosis	Diagnosis
Cutaneous mastocytosis - Maculopapular cutaneous mastocytosis ^a^ - Diffuse cutaneous mastocytosis - Mastocytoma of skin	- >15 mast cells (MCs) aggregating or more than 20 MCs per high- power field microscopy ×(40) in skin biopsy - Absence of systemic mastocytosis (SM) criteria
SM - Indolent SM (with or without skin involvement) - Smoldering SM - Aggressive SM - SM with an associated hematological neoplasm - MC leukemia	- SM criteria - Absence of C-findings ^b^ and other clonal hematological diseases -<20% of MCs in bone marrow (BM) sections - SM criteria - Absence of C-findings ^b^ and other clonal hematological diseases but two or more B-findings ^b^ - SM criteria - C-findings ^b^ - SM criteria - Demonstration of a clonal hematological non-MC disease - SM criteria - >20% of MCs in BM sections
MC sarcoma	- Infiltration of an extracutaneous organ by undifferentiated MCs with a destructive growth pattern

^a^Formerly known as urticaria pigmentosa. Main type of cutaneous mastocytosis ^82^. ^b^B-findings include (i) infiltration grade (MC) in BM of more than 30% and serum tryptase of more than 200 ng/mL, (ii) dysmyelopoiesis, and (iii) organomegaly without impariment of organ function ^4^. C-findings indicate organ dysfunction due to widespread MC infiltration, including cytopenias, osteolysis, malabsorption, and organomegaly with functional impairment of the organ/tissue (hypersplenism, portal hypertension, ascites) ^4^. Diagnostic criteria: At least one major criterion and one minor criterion or at least three minor criteria must be fulfilled for the diagnosis of SM to established. Major diagnostic criteria: multifocal dense infiltrates of MCs (>15 MCs aggregating) detected in BM sections and/or other extracutaneous organ(s) by tryptase immunohistochemistry or other MC-associated stains. Minor diagnostic criteria: (1) more than 25% of MCs are spindle-shaped in MC infiltrates detected in BM sections or other extracutaneous tissue sections OR of more than 25% atypical MCs (type I plus type II) detected in BM smears; (2) detection of a *KIT* point mutation at codon 816 in BM MCs or other extracutaneous organ(s); (3) expression of CD25 or CD2 (or both) on MCs in BM MCs, blood, or other extracutaneous tissues; (4) total serum baseline tryptase concentration persistently more than 20 ng/mL (in case of an associated hematologic non-MC lineage disease, this criterion is not valid).

### Indolent systemic mastocytosis

Based on previous reports in the largest series of patients, indolent systemic mastocytosis (ISM) comprises around 80% of all SM cases ^13^. Among them, around 20% of patients lack skin lesions at presentation (ISMs ^−^) ^14^. Despite the great relevance and efficiency of the WHO criteria for the diagnosis of SM, in ISMs ^−^, MCs represent only a very small proportion of all nucleated BM cells (usually fewer than 10 ^−3^ BM MCs, as assessed by flow cytometry) ^15^, and BM MC aggregates are frequently (around 30% of cases) not found in such patients with SM ^15^, in the absence of significantly increased serum baseline tryptase levels (<20 μg/L). Consequently, the use of highly sensitive and specific methodological approaches to the study of BM MCs becomes critical in order to avoid a misdiagnosis in patients presenting with low tumor burden ^16^.

### Mast cell activation syndromes

The term MC activation syndrome (MCAS) encompasses a heterogeneous group of diseases which are characterized by systemic symptoms secondary to MC mediator release that (i) might or might not have a known trigger, (ii) might or might not be associated with immunoglobulin E (IgE)-specific antibodies in response to that trigger, (iii) are associated with normal or elevated baseline tryptase levels, and (iv) do not show skin lesions of mastocytosis ^17^. In Table 2, the most frequent and relevant clinical symptoms suggesting an underlying MCAS are listed, and Table 3 depicts the diagnostic criteria for MCAS.

**Table 2.** Main symptoms and signs associated with the release of mast cell mediators which are considered to substantially contribute to the clinical manifestation of mast cell activation syndrome.

Mediator	Symptoms and signs
Histamine	Headache, hypotension, urticaria with or without angioedema, pruritus, diarrhea
Tryptase	Endothelial activation with associated inflammatory reaction
Chymase	Hypertension, arrythmia
Proteoglycan (heparin)	Bleeding diathesis
Platelet-activating factor	Abdominal cramping, pulmonary edema, urticaria, bronchoconstriction, hypotension, arrythmia
Prostaglandin D2	Mucus secretion, bronchoconstriction, vascular instability
LTC4 and LTD4	Mucus secretion, edema formation, vascular instability
Proinflammatory cytokines	Local inflammation, edema formation, leukocyte migration
Chemokines	Acute inflammation and leukocyte recruitment, leukocyte migration

LT, leukotriene. Adapted with permission from Karger ^17^.

**Table 3.** Criteria for the diagnosis of mast cell activation syndrome ^17^.

Criteria
1. Typical clinical symptoms ^a^
2. Increase in serum total tryptase by at least 20% above baseline plus 2 ng/mL during or within 4 hours after a symptomatic period
3. Response of clinical symptoms to histamine receptor ^b^ blockers or “mast cell- targeting” agents (for example, cromolyn)

^a^Different clinical symptoms are suggestive of systemic mast cell activation syndrome (MCAS). The following reached a consensus level above 70% ^17^: flushing, pruritus, urticaria, angioedema, nasal congestion, nasal pruritus, wheezing, throat swelling, headache, hypotension, and diarrhea. None of them *per se* is specific for MCAS and thus can count as MCAS criteria only in the context of the other two criteria. ^b^Histamine receptor blockers: H1 ± H2 inverse agonists Reproduced with permission from Karger ^17^

The current classification of MCAS is shown in Table 4. Based on the experience of the Spanish Network of Mastocytosis (REMA), the most relevant objective criteria to subclassify MCAS rely on the presence versus absence of clonal MCs as defined by the expression of CD25 (for example, CD25 ^+^ versus CD25 ^−^) or a *KIT* mutation, particularly *KIT* D816V, or both. When MCAS diagnostic criteria are fulfilled but there is no evidence of clonality, non-clonal-MCAS should be considered and co-existence of allergy or other underlying diseases should be confirmed or ruled out ^18^.

**Table 4.** Classification of mast cell activation syndrome ^17^.

Diagnostic categories and variants	Proposed criteria
Primary mast cell activation syndrome (MCAS)	MCAS and clonality criteria are met (CD25 ^+^ or *KIT* D816V mutated MCs or both) ^a^
Mastocytosis	
Clonal or monoclonal MCAS (c-MCAS)	
Secondary MCAS	MCAS, allergy, or other mast cell (MC)- activating diseases criteria are met
Allergy	
Other underlying diseases ^b^	
Idiopathic ^c^ MCAS	MCAS criteria are met but the diagnosis of the disease that explains MC activation is not achieved

^a^CD25 ^+^ *KIT* D816V mutated MC or *KIT* D816V mutated MCs without CD25 ^+^ expression ^b^Includes autoimmune diseases, bacterial infections, and drug adverse reactions ^c^This is an exclusion diagnosis and therefore a complete study is needed in order to discard any known disease that might cause MC activation Reproduced with permission from Karger ^17^

## Clinical features of patients with primary mast cell activation syndromes: mastocytosis and clonal mast cell activation syndrome

Symptoms due to the release of MC mediators upon MC activation might be present in every category of MCAS, including mild, severe, or even life-threatening symptoms such as pruritus, flushing, gastrointestinal complaints (abdominal pain or diarrhea), cognitive symptoms, and even anaphylaxis ^19, 20^.

In indolent systemic mastocytosis with skin lesions (ISMs ^+^), MC activation symptoms are typically heterogeneous and might vary from recurrent anaphylaxis ^21, 22^ to occasional symptoms triggered by a varying number of different stimuli linked to the MC mediator release episodes ( Table 5). In turn, ISMs ^−^ are frequently characterized by serious episodes of MC mediator release triggered by different factors—for example, mainly insect sting, drugs, and foods—or they might be idiopathic ^23^; in both situations, such episodes are significantly associated with the presence of anaphylaxis with cardiovascular or vascular collapse symptoms, in the absence of both urticaria and angioedema ^23^. Early studies by the REMA have demonstrated that ISMs ^−^ patients present unique features that distinguish them from ISMs ^+^ cases ^23^: (i) a higher prevalence of men versus women, (ii) a lower frequency of symptoms outside of acute episodes, (iii) lower BM MC burden, and (iv) the presence of the *KIT* mutation usually restricted to the MC lineage ^23, 24^. Of note, such unique disease features are even more characteristic within ISMs ^−^ patients whose symptoms are triggered exclusively by insect stings, whereas ISMs ^−^ patients with other triggering factors show clinical characteristics at presentation which are more similar to those of ISMs ^+^ cases ^24^.

**Table 5.** Triggers of mast cell mediator release in mast cell activation syndrome and recommendations of avoidance.

Trigger	Recommendations
Physical agents	
- Heat, changes in temperature	- Use air conditioning when necessary and mildly warm water for bath/ shower
- Friction on mastocytomas	- Avoid Darier’s sign
- Manipulation of the GI system (for example, during surgery)	- Consider prophylactic anti-mediator therapy
Emotional factors	
- Stress, anxiety	- Consider anxiolytics or relaxation techniques or both
Drugs	
- NSAIDs ^a^	- Use drugs with known tolerance for each case and consider drug challenge testing whenever tolerance is unknown and the drug required
- Opioids	- Use drugs with known tolerance for each case and consider drug challenge testing whenever tolerance is unknown and the drug required
- Anesthetics ^b, c^	- Use drugs with known tolerance for each case and consider prophylactic anti-mediator therapy and anesthetic drugs with the safest profile
- Radiological contrast media ^c^	- Use contrast media with known tolerance for each case and consider prophylactic anti-mediator therapy and use low-molecular-weight contrast agents
- Interferon α2b	- Consider prophylactic anti-mediator therapy before first doses
- Cladribine ^d^	- Consider prophylactic anti-mediator therapy before first doses
- Vaccines ^c, e^	- Consider prophylactic anti-mediator therapy
- Dextrans	- Use low-molecular-weight dextran or alternative solutions
Insect sting and bites	
- Hymenoptera	- Use insect repellents; avoid perfumed lotions; wear light-colored clothes, long-sleeved shirts, and long pants; avoid going barefoot; patients with history of previous insect-induced anaphylaxis must carry an emergency kit; specific immunotherapy for IgE-mediated hymenoptera venom allergy is recommended
*- Hippobosca equina*, mosquito ^f^	- Use insect repellents; avoid perfumed lotions; wear light-colored clothes, long-sleeved shirts, and long pants; avoid going barefoot; patients with history of previous insect-induced anaphylaxis must carry an emergency kit

^a^Frequency of mast cell (MC) mediator-related symptoms of 2% in pediatric mastocytosis and 14% in adult mastocytosis ^83^ ^b^Frequency of MC mediator-related symptoms and anaphylaxis of 2% and 0.4% in adult mastocytosis, respectively ^84^; 4% of MC mediator-related symptoms and 0–2% of anaphylaxis in pediatric mastocytosis ^84– 86^ ^c^Prophylactic anti-mediator therapy is recommended in all cases ^87^ ^d^Infrequent, based on one case report (Javed Sheik, Beth Israel Hospital, Harvard Medical School, personal communication, September 2002) ^e^Infrequent, based on case report ^88^ ^f^Infrequent, based on case reports ^89, 90^ GI, gastrointestinal; IgE, immunoglobulin E; NSAID, non-steroidal anti-inflammatory drug. Adapted with permission from Ergon ^91^.

## Diagnosis of primary mast cell activation syndromes

The final diagnosis of SM and c-MCAS systematically requires a BM study for the evaluation of all disease characteristics used for the diagnosis of SM, such as BM MC cytology ^25^, histology and immunochemistry ^26, 27^, flow cytometry immunophenotyping using specific gating strategies for the detection of BM MCs present at low frequencies ^5– 7, 28^, and the study of *KIT* mutation in purified MCs ^9, 29^, together with a detailed clinical work-up. Usually, these studies are available only in reference centers, and therefore either the patient or the samples should be referred.

**Figure 1.**
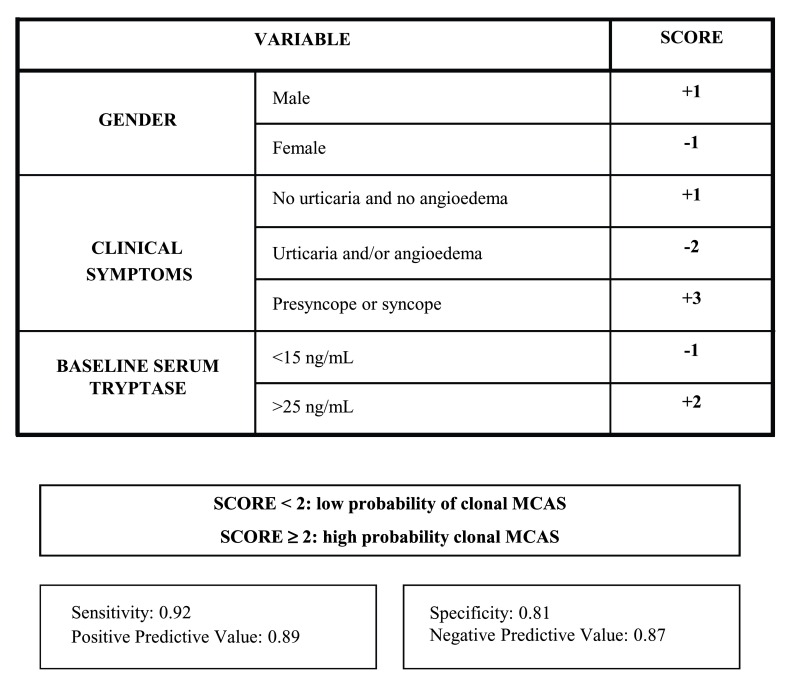
The Spanish Network on Mastocytosis score. This scoring model is proposed as a screening tool for the diagnosis of clonal mast cells in patients presenting with anaphylaxis in the absence of skin mastocytosis before a bone marrow study is performed. MCAS, mast cell activation syndrome. Reproduced with permission from Elsevier Inc. ^23^.

The European Competence Network on Mastocytosis recommends using the REMA score ( Figure 1) as a clinically useful tool to predict for the presence of clonal MCs prior to a BM study ^23^; the REMA score is based only on demographic data (gender), the symptoms and signs observed during the acute episodes, and serum baseline tryptase levels. A REMA score of at least 2 predicts with a high sensitivity and specificity for ISMs ^−^ (or c-MCAS), whereas a REMA score of less than 2 usually indicates non-clonal disease. Whether “non-clonal disease” means a true absence of any mutations whatsoever or simply the absence of clonality currently detectable in the clinical laboratory remains unclear. The REMA score is a particularly helpful tool since (i) it is based on clinical data and can be used on a routine clinical basis, (ii) it is associated with rather low costs, and (iii) it avoids unnecessary BM studies.

## Approach to diagnosis of primary mast cell activation syndromes

**Figure 2.**
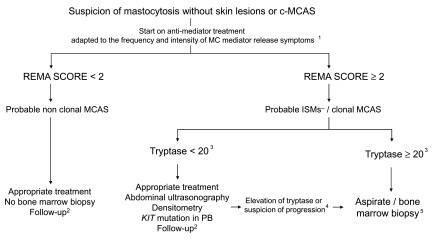
Updated algorithm proposed by REMA for the management of MCAS patients suspected of having mastocytosis without skin lesions and c-MCAS. Clonality in this figure (as in Figure 1) is limited to positivity for KIT-D816V by polymerase chain reaction or positivity for co-expression by flow cytometry of CD117 with CD25 or CD2 or both. ^1^In asymptomatic patients, only sodium cromoglicate. Depending on additional symptoms, assess adding other anti-mediator treatments. ^2^Periodic determination of tryptase together with follow-up of clinical evolution and, if necessary, image tests. ^3^Tryptase values are approximate and are based on the fact that, in patients with low values, the percentage of MCs in bone marrow is very low and therefore the possibility of finding aggregates or identifying MCs is more complicated. ^4^In cases where there is a rising trend in baseline tryptase values, clinicians are advised to wait until it rises above 20 ng/mL, at which point the probability of obtaining a sample that is suitable for conducting the study increases. The unique situation in which a bone marrow biopsy can be assessed in patients with a score of at least 2 and baseline tryptase levels of less than 20 ng/mL occurs when the patient has presented anaphylaxis following a hymenoptera sting and is a candidate for immunotherapy, given that patients with mastocytosis or c-MCAS (or both) have a greater risk of having adverse reactions during the administration of venom immunotherapy. ^5^The bone marrow study should be done only if the mentioned methods (see text, section “Diagnosis of primary mast cell activation syndromes”), and flow cytometry and cell purification in particular, are available. If the technology needed to conduct these studies is not available, it is recommended that patients be referred to specialized reference centers. c-MCAS, clonal mast cell activation syndrome; ISM ^−^, indolent systemic mastocytosis without skin lesions; MC, mast cell; MCAS, mast cell activation syndrome; PB, peripheral blood; REMA, Spanish Network on Mastocytosis. Adapted with permission from Ergon ^91^.

Whenever a patient is suspected of having a primary MCAS (either mastocytosis without skin involvement or c-MCAS), the use of the REMA score mentioned above is recommended ^23^ as an initial screening measure ( Figure 2). Patients with a REMA score of less than 2 will have a low probability of presenting clonal MCs; therefore, in such cases, it is usually not necessary to conduct additional studies other than controlling the symptoms with adequate medication and scheduling periodic follow-up visits. In contrast, patients with a REMA score of at least 2 have a high probability of presenting mastocytosis or c-MCAS. Thus, in the latter patients, it is recommended that appropriate treatment be started and that the patient be evaluated in detail for major complications of the disease, such as hepatomegaly/splenomegaly, osteopenia, and osteoporosis. To establish the most appropriate timing to perform a BM biopsy to arrive at a firm diagnosis, it might be useful to evaluate the presence of the D816V *KIT* mutation in peripheral blood (it is typically positive in approximately 80% of primary MCAS cases ^12^) and to monitor baseline serum tryptase levels and wait until they rise above 20 ng/mL, at which point the probability of obtaining a BM sample that is suitable for demonstrating BM involvement increases. Independently of the time at which the BM study is scheduled, it is recommended that BM MCs be purified prior to molecular studies, which have demonstrated greater sensitivity for the detection of *KIT* mutations ^9, 23, 30^. As an exception, BM biopsy studies might be performed in suspicious patients with a REMA score of at least 2 and baseline tryptase levels of less than 20 ng/mL who presented with anaphylaxis following a hymenoptera sting and who are candidates for immunotherapy, since patients with mastocytosis or c-MCAS (or both) have a greater risk of having adverse reactions during administration of venom immunotherapy (VIT) ^14^ (see the ‘Hymenoptera venom immunotherapy’ section below). A new quantitative real-time PCR test for KIT-D816V that is substantially more sensitive than standard KIT-D816V PCR testing has recently been developed, as the quantitative reverse transcription-PCR (qrt-PCR) test is showing essentially 100% sensitivity ^31^. Unfortunately, the availability of the qrt-PCR test at present is far more limited than that of the standard PCR test.

## Treatment of primary mast cell activation syndromes

Patients with c-MCAS or mastocytosis may present symptoms due to the release of MC mediators, associated or not with symptoms related to tissue infiltration by clonal MC (more frequent among aggressive forms of mastocytosis). At present, there is no curative therapy for mastocytosis and the treatment strategies are focused on controlling the frequency and intensity of symptoms secondary to the release of MC mediators; this includes both strict avoidance of triggers ( Table 5) and the anti-mediator therapy carefully selected on the basis of the intensity or severity (or both) of the signs and symptoms linked with the activation of MCs. Cytoreductive therapy and targeted therapies with tyrosine kinase inhibitors might be needed in selected cases presenting with elevated MC burden or aggressive and poor prognosis-associated forms of SM or patients who are unresponsive to conventional anti-mediator therapy ^32^, but these therapies will not be discussed further in this review.

### Avoidance of triggers and counseling

Careful counseling must be given to patients, caretakers, and their physicians to avoid triggers that evoke MC mediator release ^32, 33^ ( Table 5). In addition, detailed training to manage MC activation-associated episodes should be provided to patients as well as their physicians.

### Anti-mediator therapy for primary mast cell activation disorders

Anti-mediator therapy for primary MCAS aims at inhibiting production, interfering with release, blocking the specific receptors, or antagonizing the effects of MC mediators. It is used both to treat and to prevent acute and chronic MC mediator release-associated symptoms ^32^. The specific anti-mediator administration schedule (on demand or continuous administration) should be carefully selected for the individual patient on the basis of the intensity or severity (or both) of the signs and symptoms observed during the most severe acute episodes or anaphylaxis as well as the MC mediator-related symptoms recorded between acute episodes ^19^.

***Histamine receptor blockers.*** The biologic effects of histamine released from MCs, through its binding to histamine receptors (mainly H1 and H2 histamine receptors), include (i) increased vascular permeability, (ii) vasodilatation, (iii) contraction of non-vascular smooth muscle, (iv) increase of exocrine gland secretion, and (v) stimulation of the peripheral nervous system; these effects result in symptoms such as pruritus, urticaria, edema, bronchoconstriction, gastric hypersecretion, abdominal cramping, diarrhea, headache, hypotension, and anaphylaxis ^34^. The effects of H1 blockers are described in Table 6.

**Table 6.** Antimediator therapy used to control for MC mediators related symptoms: drugs most frequently used, their mechanisms of action and controlled symptoms.

Drug	Mechanism of action	Controlled symptoms
H1 antihistamines	Histamine receptor blocker	Pruritus, flushing, urticaria, swelling, tachycardia, abdominal pain related with MC degranulation processes, hypotension or reduction of the severity of symptoms of anaphylaxis ^32, 34, 42, 92, 93^
H2 antihistamines	- Histamine receptor blocker that can potentiate the effect of H1 antihistamines	Gastric hypersecretion, abdominal pain, diarrhea, and recurrent/severe MC mediator release episodes ^32, 40, 42, 94, 95^
Sodium cromol	- Unclear - Inhibits GTP-g-S-induced exocytosis in MCs and modulates sensory nerve function ^106, 107^	Abdominal pain, vomiting, diarrhea (based on double- blind placebo-controlled trials), pruritus, flushing, headache, cognitive and skeletal symptoms ^40, 45, 96– 105^
Aspirin and NSAIDs	Inhibition of cyclooxygenase and blockade of the synthesis of PGD2	Flushing, dizziness, and gastrointestinal symptoms ^48, 108^
Montelukast	Antagonizes cysLT receptor 1	Respiratory, cutaneous, gastrointestinal, and urinary symptoms ^47, 109– 111^
Zileuton	Blockade of the synthesis of LTs by inhibiting LO	Neuropsychiatric and constitutional subjective symptoms
Glucocorticoids	- Binding the intracellular glucocorticoid receptor and modulation of the transcription mediator of numerous genes - Decreased number of connective tissue MCs - Inhibition of the production of stem cell factor production and other interleukins and eicosanoid mediators - Decreased expression of chemokine receptors (for example, CCR3) - Decreased MC activation via induction of decreased expression of FcεRI - Inhibition of signaling cascades in MCs through the expression of phosphatases that are upregulated by glucocorticoids ^42, 114^	Gastrointestinal malabsorption, abdominal pain, ascites, bone disease (including diffuse bone sclerosis); acute and/or severe MC release ^40, 112, 113^
Omalizumab	Blocks the binding of IgE to the FcεRI receptor on the surface of MCs and basophils, reducing receptor expression ^123^ and the release of mediators ^124^	Cutaneous symptoms ^115– 117^, gastrointestinal symptoms ^118, 119^, anaphylaxis ^118, 120– 122^, and reactions to venom immunotherapy ^58^

GTP-g-S, guanosine triphosphate-gamma-S; IgE, immunoglobulin E; LO, lipoxygenase; LT, leukotriene; MC, mast cell; NSAID, non-steroidal anti-inflammatory drug; PGD2, prostaglandin D2.

Some H1 antihistamines such as desloratadine and ketotifen have MC-stabilizing properties, and therefore they might also decrease the release of MC mediators ^35, 36^. Ketotifen has been reported to be effective in the treatment of bone disease as well as cutaneous, gastrointestinal, and neuropsychiatric symptoms in mastocytosis ^37– 39^. Patients with associated depression might benefit from doxepin because of its effects as an antidepressant and H1 histamine blocker ^40^. Rupatadine also has an antagonistic effect against platelet-activating factor, a lipid-derived mediator which is newly synthesized and released by MCs upon their activation, resulting in hypotensive episodes and flushing ^41^.

Some patients with mastocytosis require a combination of different H1 blockers to achieve a good control of symptoms ^32^. The use of non-sedating H1 blockers is recommended for patients who require daily maintenance therapy; in turn, the use of sedating H1 antihistamines that have a fast-acting effect which makes them suitable drugs to treat acute MC mediator release episodes, frequently in association with corticosteroids or epinephrine or both, and also to prevent MC degranulation during risk situations might be administered at night or on demand ^32, 42^.

***Cromolyn sodium.*** Cromolyn sodium is an MC stabilizer that has been proven to inhibit MC activation and MC release of mediators both *in vitro* and *in vivo* despite its limited systemic absorption following ingestion; this suggests an inability of the drug to enter cells and the need for a potential interaction with an as-yet-unidentified cell surface receptor to induce its biologic activity ^43^ ( Table 6). Side effects include headache, sleepiness, irritability, abdominal pain, diarrhea, and constipation, most of which are attenuated by progressive introduction of the drug ^44^. In addition, 0.21–4% cromolyn sodium water-soluble creams, as well as aqueous-based skin lotion, may be effective at improving cutaneous symptoms (for example, pruritus and flaring of lesions) ^32, 34, 45, 46^.

***Antagonists of arachidonic acid metabolites.*** MCs synthesize *de novo* mediators and release arachidonic acid metabolites through the action of lipoxygenase and cyclooxygenase enzymes; thus, they produce leukotrienes and prostaglandins (PGs), respectively. Prostaglandin D2 is generated almost entirely by MCs and rapidly converted into active metabolites of prolonged activity rather than the parent compound, such as α11β-PGF2; the two PGs share a biological activity and induce bronchoconstriction, increase of vascular permeability, and vasodilatation, and at the same time they have chemoattractive properties for eosinophils, basophils, and Th2 lymphocytes ^47^ and are involved in the development of flushing and possibly also hypotensive episodes in patients with c-MCAS and mastocytosis ^48^ ( Table 6).

***Aspirin.*** Non-steroidal anti-inflammatory drugs, especially aspirin, can also inhibit the activation of MCs and their degranulation in some patients with mastocytosis; therefore, this therapy is recommended in patients with known tolerance to these drugs.

***Glucocorticoids.*** The mechanism of action and the effects of glucocorticoids are shown in Table 6. Short cycles of low doses of either prednisone (0.3 mg/kg per day) or oral budesonide (0.1 mg/kg per day) may improve abdominal pain refractory to treatment with cromolyn. Topical corticosteroids may be used for patients who present with skin symptoms, especially among cases with limited skin involvement; however, evidence supporting their topical use is either anecdotal or based on small series of patients ^49^.

***Anti-immunoglobulin E therapy.*** Successful anti-IgE therapy has been documented in some conditions such as severe persistent allergic asthma, chronic urticaria ^50^, idiopathic anaphylaxis ^51^, and mastocytosis ( Table 6). Until more information based on clinical trials becomes available, omalizumab in MC diseases should be restricted to selected patients with severe symptoms which have proven unresponsive to intensive anti-mediator therapy ^33^.

***Hymenoptera venom immunotherapy.*** Specific VIT is recommended for mastocytosis and c-MCAS patients with IgE-mediated anaphylaxis to hymenoptera venom; however, it should be managed as a high-risk procedure. VIT has proven effective and safe in these patients ^52– 56^; it has a rate of protection from re-stings of 86%, and the frequency of systemic reactions to VIT ranges from 5–25% ^14, 57^, most of which (75%) were associated with rush inductions (versus 25% using conventional induction) ^57^. Whenever adverse reactions to VIT prevent the protective maintenance dose of 100 μg per month from being reached, prophylactic anti-mediator therapy, changes in the extract, and administration of omalizumab therapy may be useful ^57, 58^. Furthermore, in patients who present anaphylaxis after re-sting, despite the administration of a standard maintenance dose, it is recommended that maintenance doses be increased to 200 μg ^57^. An extended maintenance administration (even lifelong) is proposed ^14, 57^ since cases presenting with fatal reactions after discontinuation of VIT have been described ^59, 60^.

### Other therapies

***Ultraviolet irradiation.*** *In vitro* studies demonstrated that long-wave ultraviolet radiation (psoralen plus ultraviolet A or ultraviolet A alone) and narrowband ultraviolet B phototherapy irradiation interfere with the release of histamine from skin-activated MCs and induce MC apoptosis ^61– 63^; all of them were employed as a second-line therapy to treat cutaneous symptoms (pruritus, whealing, and flare reactions) in patients with typical mastocytosis skin lesions who were not responsive to first-line therapies with anti-mediator drugs ^40, 64– 66^. There is no information regarding whether these therapies are useful in ISMs ^−^ and c-MCAS cases. In addition, fading of hyperpigmented skin lesions (frequently temporary) can be observed in some cases. Furthermore, responses of life-threatening MC mediator release episodes in children with bullous diffuse cutaneous mastocytosis have been reported ^67^.

***Treatment of bone mass loss.*** This is a frequent finding in both ISMs ^+^ and advanced mastocytosis but is less frequently observed among primary MCAS cases, particularly ISMs ^−^ and c-MCAS. Fractures are usually developed as a consequence of severe osteoporosis or large osteolytic lesions. Local MC infiltration and disturbances in bone remodeling which are due to the release of MC mediators such as interleukin-6 (IL-6), histamine, heparin, receptor activator of nuclear factor kappa-B ligand, osteoprotegerin, or sclerostin are involved in the pathogenesis of bone manifestations in mastocytosis ^68, 69^. Calcium and vitamin D supplements, combined with bisphosphonates, are usually the first choice for osteopenia and osteoporosis, respectively ^70– 72^. Other therapies such as estrogen replacement in postmenopausal women and denosumab or interferon-alpha ^73, 74^ in patients with severe osteoporosis at risk of pathologic bone fractures unresponsive to conventional treatments might also be considered. Regarding peptidergic/peptidomimetic drugs such as teriparatide, caution is urged since these drugs have been associated with driving MC activation via MRGPRX2 ^75^.

***Selective serotonin reuptake inhibitors.*** MCs contain numerous mediators, including neurotransmitters (for example, serotonin), cytokines, and chemokines, that play a role in stress response, behavior, and emotion regulation ^76, 77^. Furthermore, the elevation of the circulating levels of tumor necrosis factor-alpha and IL-6 with, for example, endotoxin leads to depressive symptoms, and it has been previously described that some selective serotonin reuptake inhibitors (SSRIs) such as citalopram can reduce endotoxin-induced symptoms ^78^. Based on this information, together with their good tolerability profile, SSRIs have emerged as an option to improve symptoms of depression in patients with mastocytosis ^79^.

### Other conditions

With regard to pregnancy, two different series of women with mastocytosis have been reported in the literature ^80, 81^. The larger series ^81^ included 45 pregnancies and deliveries in women with non-aggressive categories of the disease. Based on their results, anti-mediator therapy (dexchlorpheniramine, loratadine, cetirizine, ranitidine, oral corticosteroids, and adrenaline), if required during pregnancy, as well as systematic administration of prophylactic anti-mediator therapy at the beginning of labor based on drugs with a good well-known safety profile, is recommended ^81^.

## Abbreviations

BM, bone marrow; c-MCAS, clonal mast cell activation syndrome; IgE, immunoglobulin E; IL, interleukin; ISM, indolent systemic mastocytosis; MC, mast cell; MCAS, mast cell activation syndrome; PCR, polymerase chain reaction; PG, prostaglandin; qrt-PCR, quantitative reverse transcription-polymerase chain reaction; REMA, Spanish Network on Mastocytosis; SM, systemic mastocytosis; SSRI, selective serotonin reuptake inhibitor; VIT, venom immunotherapy; WHO, World Health Organization.

## References

[1] Fukuoka Y Xia H Sanchez-Muñoz LB: Generation of anaphylatoxins by human beta-tryptase from C3, C4, and C5. *J Immunol.* 2008;180(9):6307–16. 10.4049/jimmunol.180.9.6307 18424754 PMC2645414

[2] Galli SJ Dvorak AM Dvorak HF: Basophils and mast cells: morphologic insights into their biology, secretory patterns, and function. *Prog Allergy.* 1984;34:1–141. 10.1159/000318367 6230674

[3] Metzger H: The high affinity receptor for IgE on mast cells. *Clin Exp Allergy.* 1991;21(3):269–79. 10.1111/j.1365-2222.1991.tb01658.x 1830826

[4] Arber DA Orazi A Hasserjian R: The 2016 revision to the World Health Organization classification of myeloid neoplasms and acute leukemia. *Blood.* 2016;127(20):2391–405. 10.1182/blood-2016-03-643544 27069254

[5] Orfao A Escribano L Villarrubia J: Flow cytometric analysis of mast cells from normal and pathological human bone marrow samples: identification and enumeration. *Am J Pathol.* 1996;149(5):1493–9. 8909239 PMC1865253

[6] Escribano L Navalón R Nuñez R: Immunophenotypic analysis of human mast cells by flow cytometry.In: J. Robinson, Z. Darzynkiewicz, P. Dean, A. Orfao, P. Rabinovich and L. Wheeless, eds. *Curr Protoc Cytom.*New York: John Wiley & Sons, Inc;2001; Chapter 6: Unit 6.6. 10.1002/0471142956.cy0606s12 18770717

[7] Escribano L Diaz-Agustin B López A: Immunophenotypic analysis of mast cells in mastocytosis: When and how to do it. Proposals of the Spanish Network on Mastocytosis (REMA). *Cytometry B Clin Cytom.* 2004;58(1):1–8. 10.1002/cyto.b.10072 14994369

[8] Teodosio C García-Montero AC Jara-Acevedo M: Mast cells from different molecular and prognostic subtypes of systemic mastocytosis display distinct immunophenotypes. *J Allergy Clin Immunol.* 2010;125(3):719–26,726.e1–726.e4. 10.1016/j.jaci.2009.10.020 20061010

[9] Garcia-Montero AC Jara-Acevedo M Teodosio C: *KIT* mutation in mast cells and other bone marrow hematopoietic cell lineages in systemic mast cell disorders: a prospective study of the Spanish Network on Mastocytosis (REMA) in a series of 113 patients. *Blood.* 2006;108(7):2366–72. 10.1182/blood-2006-04-015545 16741248

[10] Kristensen T Vestergaard H Møller MB: Improved detection of the *KIT* D816V mutation in patients with systemic mastocytosis using a quantitative and highly sensitive real-time qPCR assay. *J Mol Diagn.* 2011;13(2):180–8. 10.1016/j.jmoldx.2010.10.004 21354053 PMC3279709

[11] Kristensen T Vestergaard H Bindslev-Jensen C: Sensitive *KIT* D816V mutation analysis of blood as a diagnostic test in mastocytosis. *Am J Hematol.* 2014;89(5):493–8. 10.1002/ajh.23672 24443360

[12] Jara-Acevedo M Teodosio C Sanchez-Muñoz L: Detection of the *KIT* D816V mutation in peripheral blood of systemic mastocytosis: diagnostic implications. *Mod Pathol.* 2015;28(8):1138–49. 10.1038/modpathol.2015.72 26067933

[13] Escribano L Garcia-Montero A Sanchez-Muñoz L: Diagnosis of Adult Mastocytosis: Role for Bone Marrow Analysis. In: K. Kottke-Marchant and B. Davis, eds. *Laboratory Hematology Practice* London: Wiley-Blackwell;2012;388–98. 10.1002/9781444398595.ch29

[14] González-de-Olano D Alvarez-Twose I Vega A: Venom immunotherapy in patients with mastocytosis and hymenoptera venom anaphylaxis. *Immunotherapy.* 2011;3(5):637–51. 10.2217/imt.11.44 21554093

[15] Escribano L Orfao A Villarrubia J: Sequential immunophenotypic analysis of mast cells in a case of systemic mast cell disease evolving to a mast cell leukemia. *Cytometry.* 1997;30(2):98–102. 10.1002/(SICI)1097-0320(19970415)30:2<98::AID-CYTO4>3.0.CO;2-9 9149916

[16] Sánchez-Muñoz L Morgado JM Álvarez-Twose I: Diagnosis and classification of mastocytosis in non-specialized *versus* reference centres: a Spanish Network on Mastocytosis (REMA) study on 122 patients. *Br J Haematol.* 2016;172(1):56–63. 10.1111/bjh.13789 26456532

[17] Valent P Akin C Arock M: Definitions, criteria and global classification of mast cell disorders with special reference to mast cell activation syndromes: a consensus proposal. *Int Arch Allergy Immunol.* 2012;157(3):215–25. 10.1159/000328760 22041891 PMC3224511

[18] Cardet JC Castells MC Hamilton MJ: Immunology and clinical manifestations of non-clonal mast cell activation syndrome. *Curr Allergy Asthma Rep.* 2013;13(1):10–8. 10.1007/s11882-012-0326-8 23212667 PMC3545645

[19] Matito A Alvarez-Twose I Morgado JM: Anaphylaxis as a clinical manifestation of clonal mast cell disorders. *Curr Allergy Asthma Rep.* 2014;14(8):450. 10.1007/s11882-014-0450-8 24947681

[20] Valent P Escribano L Broesby-Olsen S: Proposed diagnostic algorithm for patients with suspected mastocytosis: a proposal of the European Competence Network on Mastocytosis. *Allergy.* 2014;69(10):1267–74. 10.1111/all.12436 24836395

[21] Brockow K Jofer C Behrendt H: Anaphylaxis in patients with mastocytosis: a study on history, clinical features and risk factors in 120 patients. *Allergy.* 2008;63(2):226–32. 10.1111/j.1398-9995.2007.01569.x 18186813

[22] González de Olano D de la Hoz Caballer B Núñez López R: Prevalence of allergy and anaphylactic symptoms in 210 adult and pediatric patients with mastocytosis in Spain: a study of the Spanish network on mastocytosis (REMA). *Clin Exp Allergy.* 2007;37(10):1547–55. 10.1111/j.1365-2222.2007.02804.x 17883734

[23] Alvarez-Twose I González de Olano D Sánchez-Muñoz L: Clinical, biological, and molecular characteristics of clonal mast cell disorders presenting with systemic mast cell activation symptoms. *J Allergy Clin Immunol.* 2010;125(6):1269–1278.e2. 10.1016/j.jaci.2010.02.019 20434205

[24] Bonadonna P Perbellini O Passalacqua G: Clonal mast cell disorders in patients with systemic reactions to Hymenoptera stings and increased serum tryptase levels. *J Allergy Clin Immunol.* 2009;123(3):680–6. 10.1016/j.jaci.2008.11.018 19135713

[25] Sperr WR Escribano L Jordan JH: Morphologic properties of neoplastic mast cells: delineation of stages of maturation and implication for cytological grading of mastocytosis. *Leuk Res.* 2001;25(7):529–36. 10.1016/S0145-2126(01)00041-8 11377677

[26] Horny HP Sillaber C Menke D: Diagnostic value of immunostaining for tryptase in patients with mastocytosis. *Am J Surg Pathol.* 1998;22(9):1132–40. 9737247 10.1097/00000478-199809000-00013

[27] Horny HP Valent P: Diagnosis of mastocytosis: general histopathological aspects, morphological criteria, and immunohistochemical findings. *Leuk Res.* 2001;25(7):543–51. 10.1016/S0145-2126(01)00021-2 11377679

[28] Escribano L Orfao A Díaz-Agustin B: Indolent systemic mast cell disease in adults: immunophenotypic characterization of bone marrow mast cells and its diagnostic implications. *Blood.* 1998;91(8):2731–6. 9531582

[29] Sotlar K Escribano L Landt O: One-step detection of c-kit point mutations using peptide nucleic acid-mediated polymerase chain reaction clamping and hybridization probes. *Am J Pathol.* 2003;162(3):737–46. 10.1016/S0002-9440(10)63870-9 12598308 PMC1868096

[30] Alvarez-Twose I Zanotti R González-de-Olano D: Nonaggressive systemic mastocytosis (SM) without skin lesions associated with insect-induced anaphylaxis shows unique features versus other indolent SM. *J Allergy Clin Immunol.* 2014;133(2):520–8. 10.1016/j.jaci.2013.06.020 23921094

[31] De Matteis G Zanotti R Colarossi S: The impact of sensitive *KIT* D816V detection on recognition of indolent Systemic Mastocytosis. *Leuk Res.* 2015;39(3):273–8. 10.1016/j.leukres.2014.11.029 25582384

[32] Alvarez-Twose I Matito A Sánchez-Muñoz L: Management of adult mastocytosis. *Expert Opin Orphan Drugs.* 2014;2(4):321–36. 10.1517/21678707.2014.884922

[33] Siebenhaar F Akin C Bindslev-Jensen C: Treatment strategies in mastocytosis. *Immunol Allergy Clin North Am.* 2014;34(2):433–47. 10.1016/j.iac.2014.01.012 24745685

[34] Arock M Akin C Hermine O: Current treatment options in patients with mastocytosis: status in 2015 and future perspectives. *Eur J Haematol.* 2015;94(6):474–90. 10.1111/ejh.12544 25753531

[35] Okayama Y Church MK: Comparison of the modulatory effect of ketotifen, sodium cromoglycate, procaterol and salbutamol in human skin, lung and tonsil mast cells. *Int Arch Allergy Immunol.* 1992;97(3):216–25. 10.1159/000236122 1375203

[36] Weller K Maurer M: Desloratadine inhibits human skin mast cell activation and histamine release. *J Invest Dermatol.* 2009;129(11):2723–6. 10.1038/jid.2009.134 19516262

[37] Graves L 3rd Stechschulte DJ Morris DC: Inhibition of mediator release in systemic mastocytosis is associated with reversal of bone changes. *J Bone Miner Res.* 1990;5(11):1113–9. 10.1002/jbmr.5650051104 2270775

[38] Póvoa P Ducla-Soares J Fernandes A: A case of systemic mastocytosis; therapeutic efficacy of ketotifen. *J Intern Med.* 1991;229(5):475–7. 10.1111/j.1365-2796.1991.tb00379.x 2040876

[39] Ting S: Ketotifen and systemic mastocytosis. *J Allergy Clin Immunol.* 1990;85(4):818. 10.1016/0091-6749(90)90205-I 2324420

[40] Escribano L Akin C Castells M: Current options in the treatment of mast cell mediator-related symptoms in mastocytosis. *Inflamm Allergy Drug Targets.* 2006;5(1):61–77. 10.2174/187152806775269303 16613565

[41] Siebenhaar F Förtsch A Krause K: Rupatadine improves quality of life in mastocytosis: a randomized, double-blind, placebo-controlled trial. *Allergy.* 2013;68(7):949–52. 10.1111/all.12159 23734572

[42] Cardet JC Akin C Lee MJ: Mastocytosis: update on pharmacotherapy and future directions. *Expert Opin Pharmacother.* 2013;14(15):2033–45. 10.1517/14656566.2013.824424 24044484 PMC4362676

[43] Edwards A Stepehen T: The Chromones: sodium cromolyn and nedocromil sodium. In: N.F. Adkinson, ed. *Middleton's allergy: principles and practice* China: Mosby;2009.

[44] Lester MR Bratton DL: Adverse reactions to cromolyn sodium: patient report and review of the literature. *Clin Pediatr (Phila).* 1997;36(12):707–10. 10.1177/000992289703601207 9415839

[45] Edwards AM Capková S: Oral and topical sodium cromoglicate in the treatment of diffuse cutaneous mastocytosis in an infant. *BMJ Case Rep.* 2011;2011. pii: bcr0220113910. 10.1136/bcr.02.2011.3910 22693187 PMC3128352

[46] Vieira Dos Santos R Magerl M Martus P: Topical sodium cromoglicate relieves allergen- and histamine-induced dermal pruritus. *Br J Dermatol.* 2010;162(3):674–6. 10.1111/j.1365-2133.2009.09516.x 19785618

[47] Bochenek G Nizankowska E Gielicz A: Plasma 9alpha,11beta-PGF2, a PGD2 metabolite, as a sensitive marker of mast cell activation by allergen in bronchial asthma. *Thorax.* 2004;59(6):459–64. 10.1136/thx.2003.013573 15170023 PMC1747024

[48] Butterfield JH: Survey of aspirin administration in systemic mastocytosis. *Prostaglandins Other Lipid Mediat.* 2009;88(3–4):122–4. 10.1016/j.prostaglandins.2009.01.001 19429499

[49] Barton J Lavker RM Schechter NM: Treatment of urticaria pigmentosa with corticosteroids. *Arch Dermatol.* 1985;121(12):1516–23. 10.1001/archderm.1985.01660120042017 4062333

[50] Kaplan AP Joseph K Maykut RJ: Treatment of chronic autoimmune urticaria with omalizumab. *J Allergy Clin Immunol.* 2008;122(3):569–73. 10.1016/j.jaci.2008.07.006 18774392

[51] Warrier P Casale TB: Omalizumab in idiopathic anaphylaxis. *Ann Allergy Asthma Immunol.* 2009;102(3):257–8. 10.1016/S1081-1206(10)60091-9 19354075

[52] Engler RJ Davis WS: Rush Hymenoptera venom immunotherapy: successful treatment in a patient with systemic mast cell disease. *J Allergy Clin Immunol.* 1994;94(3 Pt 1):556–9. 10.1016/0091-6749(94)90213-5 8083461

[53] Fricker M Helbling A Schwartz L: Hymenoptera sting anaphylaxis and urticaria pigmentosa: clinical findings and results of venom immunotherapy in ten patients. *J Allergy Clin Immunol.* 1997;100(1):11–5. 10.1016/S0091-6749(97)70188-X 9257781

[54] Haeberli G Bronnimann M Hunziker T: Elevated basal serum tryptase and hymenoptera venom allergy: relation to severity of sting reactions and to safety and efficacy of venom immunotherapy. *Clin Exp Allergy.* 2003;33(9):1216–20. 10.1046/j.1365-2222.2003.01755.x 12956741

[55] Bonadonna P Zanotti R Caruso B: Allergen specific immunotherapy is safe and effective in patients with systemic mastocytosis and *Hymenoptera* allergy. *J Allergy Clin Immunol.* 2008;121(1):256–7. 10.1016/j.jaci.2007.10.014 18206512

[56] González de Olano D Alvarez-Twose I Esteban-López MI: Safety and effectiveness of immunotherapy in patients with indolent systemic mastocytosis presenting with Hymenoptera venom anaphylaxis. *J Allergy Clin Immunol.* 2008;121(2):519–26. 10.1016/j.jaci.2007.11.010 18177694

[57] Bonadonna P Gonzalez-de-Olano D Zanotti R: Venom immunotherapy in patients with clonal mast cell disorders: efficacy, safety, and practical considerations. *J Allergy Clin Immunol Pract.* 2013;1(5):474–8. 10.1016/j.jaip.2013.06.014 24565619

[58] Kontou-Fili K Filis CI: Prolonged high-dose omalizumab is required to control reactions to venom immunotherapy in mastocytosis. *Allergy.* 2009;64(9):1384–5. 10.1111/j.1398-9995.2009.02045.x 19432937

[59] Dubois AE: Mastocytosis and Hymenoptera allergy. *Curr Opin Allergy Clin Immunol.* 2004;4(4):291–5. 10.1097/01.all.0000136756.20701.f8 15238795

[60] Oude Elberink JN de Monchy JG Kors JW: Fatal anaphylaxis after a yellow jacket sting, despite venom immunotherapy, in two patients with mastocytosis. *J Allergy Clin Immunol.* 1997;99(1 Pt 1):153–4. 10.1016/S0091-6749(97)70314-2 9003225

[61] Guhl S Stefaniak R Strathmann M: Bivalent effect of UV light on human skin mast cells-low-level mediator release at baseline but potent suppression upon mast cell triggering. *J Invest Dermatol.* 2005;124(2):453–6. 10.1111/j.0022-202X.2004.23523.x 15675967

[62] Guhl S Hartmann K Tapkenhinrichs S: Ultraviolet irradiation induces apoptosis in human immature, but not in skin mast cells. *J Invest Dermatol.* 2003;121(4):837–44. 10.1046/j.1523-1747.2003.12480.x 14632203

[63] Kronauer C Eberlein-Konig B Ring J: Influence of UVB, UVA and UVA1 irradiation on histamine release from human basophils and mast cells *in vitro* in the presence and absence of antioxidants. *Photochem Photobiol.* 2003;77(5):531–4. 10.1562/0031-8655(2003)0770531IOUUAU2.0.CO2 12812296

[64] Prignano F Troiano M Lotti T: Cutaneous mastocytosis: successful treatment with narrowband ultraviolet B phototherapy. *Clin Exp Dermatol.* 2010;35(8):914–5. 10.1111/j.1365-2230.2010.03825.x 20456394

[65] Brazzelli V Grasso V Manna G: Indolent systemic mastocytosis treated with narrow-band UVB phototherapy: study of five cases. *J Eur Acad Dermatol Venereol.* 2012;26(4):465–9. 10.1111/j.1468-3083.2011.04098.x 21564325

[66] Zandi S Kalia S Lui H: UVA1 phototherapy: a concise and practical review. *Skin Therapy Lett.* 2012;17(1):1–4. 22358227

[67] Smith ML Orton PW Chu H: Photochemotherapy of dominant, diffuse, cutaneous mastocytosis. *Pediatr Dermatol.* 1990;7(4):251–5. 10.1111/j.1525-1470.1990.tb01020.x 2080117

[68] Manolagas SC Jilka RL: Bone marrow, cytokines, and bone remodeling. Emerging insights into the pathophysiology of osteoporosis. *N Engl J Med.* 1995;332(5):305–11. 10.1056/NEJM199502023320506 7816067

[69] Rabenhorst A Christopeit B Leja S: Serum levels of bone cytokines are increased in indolent systemic mastocytosis associated with osteopenia or osteoporosis. *J Allergy Clin Immunol.* 2013;132(5):1234–1237.e7. 10.1016/j.jaci.2013.06.019 23910691

[70] Lim AY Ostor AJ Love S: Systemic mastocytosis: a rare cause of osteoporosis and its response to bisphosphonate treatment. *Ann Rheum Dis.* 2005;64(6):965–6. 10.1136/ard.2004.029116 15897317 PMC1755523

[71] Rossini M Zanotti R Viapiana O: Bone involvement and osteoporosis in mastocytosis. *Immunol Allergy Clin North Am.* 2014;34(2):383–96. 10.1016/j.iac.2014.01.011 24745681

[72] Sallés M Holgado S Navarro JT: [Osteoporosis as a first manifestation of systemic mastocytosis. Study of 6 cases]. *Med Clin (Barc).* 2007;128(6):216–8. 10.1016/S0025-7753(07)72541-1 17335726

[73] Lehmann T Beyeler C Lämmle B: Severe osteoporosis due to systemic mast cell disease: successful treatment with interferon alpha-2B. *Br J Rheumatol.* 1996;35(9):898–900. 10.1093/rheumatology/35.9.898 8810675

[74] Weide R Ehlenz K Lorenz W: Successful treatment of osteoporosis in systemic mastocytosis with interferon alpha-2b. *Ann Hematol.* 1996;72(1):41–3. 10.1007/BF00663015 8605279

[75] McNeil BD Pundir P Meeker S: Identification of a mast-cell-specific receptor crucial for pseudo-allergic drug reactions. *Nature.* 2015;519(7542):237–41. 10.1038/nature14022 25517090 PMC4359082

[76] Nautiyal KM Ribeiro AC Pfaff DW: Brain mast cells link the immune system to anxiety-like behavior. *Proc Natl Acad Sci U S A.* 2008;105(46):18053–7. 10.1073/pnas.0809479105 19004805 PMC2584714

[77] Nautiyal KM Dailey CA Jahn JL: Serotonin of mast cell origin contributes to hippocampal function. *Eur J Neurosci.* 2012;36(3):2347–59. 10.1111/j.1460-9568.2012.08138.x 22632453 PMC3721752

[78] Hannestad J DellaGioia N Ortiz N: Citalopram reduces endotoxin-induced fatigue. *Brain Behav Immun.* 2011;25(2):256–9. 10.1016/j.bbi.2010.10.013 20955776 PMC3025065

[79] Moura DS Georgin-Lavialle S Gaillard R: Neuropsychological features of adult mastocytosis. *Immunol Allergy Clin North Am.* 2014;34(2):407–22. 10.1016/j.iac.2014.02.001 24745683

[80] Worobec AS Akin C Scott LM: Mastocytosis complicating pregnancy. *Obstet Gynecol.* 2000;95(3):391–5. 10711550 10.1016/s0029-7844(99)00591-8

[81] Matito A Álvarez-Twose I Morgado JM: Clinical impact of pregnancy in mastocytosis: a study of the Spanish Network on Mastocytosis (REMA) in 45 cases. *Int Arch Allergy Immunol.* 2011;156(1):104–11. 10.1159/000321954 21447966

[82] Hartmann K Escribano L Grattan C: Cutaneous manifestations in patients with mastocytosis: Consensus report of the European Competence Network on Mastocytosis; the American Academy of Allergy, Asthma & Immunology; and the European Academy of Allergology and Clinical Immunology. *J Allergy Clin Immunol.* 2016;137(1):35–45. 10.1016/j.jaci.2015.08.034 26476479

[83] Sánchez-Matas I Matito A Gonzalez de Olano D: Prevalence of hypersensitivity reactions to nonsteroidal anti-inflamatory drugs in 212 patients with mastocytosis in Spain. *Allergy.* 2009;64(Suppl 90):551–598. 10.1111/j.1398-9995.2009.02078.x

[84] Matito A Morgado JM Sánchez-López P: Management of Anesthesia in Adult and Pediatric Mastocytosis: A Study of the Spanish Network on Mastocytosis (REMA) Based on 726 Anesthetic Procedures. *Int Arch Allergy Immunol.* 2015;167(1):47–56. 10.1159/000436969 26160029

[85] Carter MC Uzzaman A Scott LM: Pediatric mastocytosis: routine anesthetic management for a complex disease. *Anesth Analg.* 2008;107(2):422–7. 10.1213/ane.0b013e31817e6d7c 18633019 PMC2736554

[86] Ahmad N Evans P Lloyd-Thomas AR: Anesthesia in children with mastocytosis--a case based review. *Paediatr Anaesth.* 2009;19(2):97–107. 10.1111/j.1460-9592.2008.02904.x 19207896

[87] Escribano L Orfao A: Anaphylaxis in mastocytosis. In: M. Castells, ed. *Anaphylaxis and Hypersensitivity Reactions: From Molecular Markers to Rapid Desensitization* New York; Humana Press,2011; (1):257–269. 10.1007/978-1-60327-951-2_16

[88] Bankova LG Walter JE Iyengar SR: Generalized bullous eruption after routine vaccination in a child with diffuse cutaneous mastocytosis. *J Allergy Clin Immunol Pract.* 2013;1(1):94–6. 10.1016/j.jaip.2012.08.008 24229828

[89] Matito A Bartolomé-Zavala B Alvarez-Twose I: IgE-mediated anaphylaxis to *Hippobosca equina* in a patient with systemic mastocytosis. *Allergy.* 2010;65(8):1058–9. 10.1111/j.1398-9995.2009.02270.x 20002662

[90] Reiter N Reiter M Altrichter S: Anaphylaxis caused by mosquito allergy in systemic mastocytosis. *Lancet.* 2013;382(9901):1380. 10.1016/S0140-6736(13)61605-0 24139121

[91] Gonzalez de Olano D Alvarez-Twose I Castells M: Síndromes de activación mastocitaria. In: IJ Dávila González, I Jáuregui Presa, JM Olaguible Rivera and JM Zubeldia, eds. *Tratado de Alergología* 2 ed. Madrid: Ergon;2016; (4):1316–1330.

[92] Escribano L Akin C Castells M: Mastocytosis: current concepts in diagnosis and treatment. *Ann Hematol.* 2002;81(12):677–90. 10.1007/s00277-002-0575-z 12483363

[93] Worobec AS Metcalfe DD: Mastocytosis: current treatment concepts. *Int Arch Allergy Immunol.* 2002;127(2):153–5. 10.1159/000048189 11919428

[94] Hirschowitz BI Groarke JF: Effect of cimetidine on gastric hypersecretion and diarrhea in systemic mastocytosis. *Ann Intern Med.* 1979;90(5):769–71. 10.7326/0003-4819-90-5-769 373561

[95] Gasior-Chrzan B Falk ES: Systemic mastocytosis treated with histamine H _1_ and H _2_ receptor antagonists. *Dermatology.* 1992;184(2):149–52. 10.1159/000247526 1498379

[96] Dolovich J Punthakee ND MacMillan AB: Systemic mastocytosis: control of lifelong diarrhea by ingested disodium cromoglycate. *Can Med Assoc J.* 1974;111(7):684–5. 4213416 PMC1947871

[97] Soter NA Austen KF Wasserman SI: Oral disodium cromoglycate in the treatment of systemic mastocytosis. *N Engl J Med.* 1979;301(9):465–9. 10.1056/NEJM197908303010903 111124

[98] Czarnetzki BM Behrendt H: Urticaria pigmentosa: clinical picture and response to oral disodium cromoglycate. *Br J Dermatol.* 1981;105(5):563–7. 10.1111/j.1365-2133.1981.tb00800.x 6794592

[99] Welch EA Alper JC Bogaars H: Treatment of bullous mastocytosis with disodium cromoglycate. *J Am Acad Dermatol.* 1983;9(3):349–53. 10.1016/S0190-9622(83)70140-4 6415132

[100] Lindskov R Lange Wantzin G Knudsen L: Urticaria pigmentosa treated with oral disodium cromoglycate. *Dermatologica.* 1984;169(1):49–52. 10.1159/000249567 6432596

[101] Alexander RR: Disodium cromoglycate in the treatment of systemic mastocytosis involving only bone. *Acta Haematol.* 1985;74(2):108–10. 10.1159/000206179 3937412

[102] Rogers MP Bloomingdale K Murawski BJ: Mixed organic brain syndrome as a manifestation of systemic mastocytosis. *Psychosom Med.* 1986;48(6):437–47. 10.1097/00006842-198607000-00006 3749421

[103] Leaf FA Jaecks EP Rodriguez DR: Bullous urticaria pigmentosa. *Cutis.* 1996;58(5):358–60. 8934078

[104] Horan RF Sheffer AL Austen KF: Cromolyn sodium in the management of systemic mastocytosis. *J Allergy Clin Immunol.* 1990;85(5):852–5. 10.1016/0091-6749(90)90067-E 2110198

[105] Metcalfe DD: The treatment of mastocytosis: an overview. *J Invest Dermatol.* 1991;96(3):55S–56S; discussion 56S–59S. 10.1111/1523-1747.ep12469049 2002266

[106] Martin MW O'Sullivan AJ Gomperts BD: Inhibition by cromoglycate and some flavonoids of nucleoside diphosphate kinase and of exocytosis from permeabilized mast cells. *Br J Pharmacol.* 1995;115(6):1080–6. 10.1111/j.1476-5381.1995.tb15921.x 7582506 PMC1909018

[107] Norris AA: Pharmacology of sodium cromoglycate. *Clin Exp Allergy.* 1996;26(Suppl 4):5–7. 10.1111/j.1365-2222.1996.tb00661.x 8809432

[108] Moreno-Borque R Matito A Álvarez-Twose I: Response to celecoxib in a patient with indolent systemic mastocytosis presenting with intractable diarrhea. *Ann Allergy Asthma Immunol.* 2015;115(5):456–7. 10.1016/j.anai.2015.08.016 26505934

[109] Sancho-Chust JN Chiner E Camarasa A: Recent-onset bronchial asthma as a manifestation of systemic mastocytosis. *J Investig Allergol Clin Immunol.* 2009;19(6):513–5. 20128434

[110] Tolar J Tope WD Neglia JP: Leukotriene-receptor inhibition for the treatment of systemic mastocytosis. *N Engl J Med.* 2004;350(7):735–6. 10.1056/NEJM200402123500723 14960756

[111] Turner PJ Kemp AS Rogers M: Refractory symptoms successfully treated with leukotriene inhibition in a child with systemic mastocytosis. *Pediatr Dermatol.* 2012;29(2):222–3. 10.1111/j.1525-1470.2011.01576.x 22044360

[112] Hauswirth AW Simonitsch-Klupp I Uffmann M: Response to therapy with interferon alpha-2b and prednisolone in aggressive systemic mastocytosis: report of five cases and review of the literature. *Leuk Res.* 2004;28(3):249–57. 10.1016/S0145-2126(03)00259-5 14687620

[113] Matito A Morgado JM Álvarez-Twose I: Serum tryptase monitoring in indolent systemic mastocytosis: association with disease features and patient outcome. *PLoS One.* 2013;8(10):e76116. 10.1371/journal.pone.0076116 24155887 PMC3796517

[114] Oppong E Flink N Cato AC: Molecular mechanisms of glucocorticoid action in mast cells. *Mol Cell Endocrinol.* 2013;380(1–2):119–26. 10.1016/j.mce.2013.05.014 23707629

[115] Siebenhaar F Kühn W Zuberbier T: Successful treatment of cutaneous mastocytosis and Ménière disease with anti-IgE therapy. *J Allergy Clin Immunol.* 2007;120(1):213–5. 10.1016/j.jaci.2007.05.011 17544095

[116] Matito A Blázquez-Goñi C Morgado JM: Short-term omalizumab treatment in an adolescent with cutaneous mastocytosis. *Ann Allergy Asthma Immunol.* 2013;111(5):425–6. 10.1016/j.anai.2013.08.014 24125156

[117] Sokol KC Ghazi A Kelly BC: Omalizumab as a desensitizing agent and treatment in mastocytosis: a review of the literature and case report. *J Allergy Clin Immunol Pract.* 2014;2(3):266–70. 10.1016/j.jaip.2014.03.009 24811015

[118] Carter MC Robyn JA Bressler PB: Omalizumab for the treatment of unprovoked anaphylaxis in patients with systemic mastocytosis. *J Allergy Clin Immunol.* 2007;119(6):1550–1. 10.1016/j.jaci.2007.03.032 17481708

[119] Lieberoth S Thomsen SF: Cutaneous and gastrointestinal symptoms in two patients with systemic mastocytosis successfully treated with omalizumab. *Case Rep Med.* 2015;2015: 903541. 10.1155/2015/903541 25694784 PMC4324745

[120] Douglass JA Carroll K Voskamp A: Omalizumab is effective in treating systemic mastocytosis in a nonatopic patient. *Allergy.* 2010;65(7):926–7. 10.1111/j.1398-9995.2009.02259.x 19889117

[121] Paraskevopoulos G Sifnaios E Christodoulopoulos K: Successful treatment of mastocytic anaphylactic episodes with reduction of skin mast cells after anti-IgE therapy. *Eur Ann Allergy Clin Immunol.* 2013;45(2):52–5. 23821833

[122] Kibsgaard L Skjold T Deleuran M: Omalizumab induced remission of idiopathic anaphylaxis in a patient suffering from indolent systemic mastocytosis. *Acta Derm Venereol.* 2014;94(3):363–4. 10.2340/00015555-1687 24162824

[123] Beck LA Marcotte GV MacGlashan D: Omalizumab-induced reductions in mast cell FcεRI expression and function. *J Allergy Clin Immunol.* 2004;114(3):527–30. 10.1016/j.jaci.2004.06.032 15356552

[124] Oliver JM Tarleton CA Gilmartin L: Reduced FcεRI-mediated release of asthma-promoting cytokines and chemokines from human basophils during omalizumab therapy. *Int Arch Allergy Immunol.* 2010;151(4):275–84. 10.1159/000250436 19844128 PMC2853585

